# Larvae and pupae of two North American darkling beetles (Coleoptera, Tenebrionidae, Stenochiinae), *Glyptotus cribratus* LeConte and *Cibdelis blaschkei* Mannerheim, with notes on ecological and behavioural similarities

**DOI:** 10.3897/zookeys.415.6891

**Published:** 2014-06-12

**Authors:** Warren E. Steiner

**Affiliations:** 1 Department of Entomology, NHB-187, Smithsonian Institution, Washington, DC 20013-7012

**Keywords:** Antipredator defense, identification, immature stages, North America, pinching organs, rotten wood, saproxylic insects, urogomphi

## Abstract

This study describes and illustrates the larvae and pupae of two North American darkling beetles (Coleoptera: Tenebrionidae) in the subfamily Stenochiinae, *Glyptotus cribratus* LeConte from the southeastern United States, and *Cibdelis blaschkei* Mannerheim from California. Both species inhabit forested regions where adults and larvae occur in soft rotten dry wood of dead branches on living trees or in sections recently fallen from them. Species identity was confirmed by rearing of adults and pupae and the discovery of both in pupal cells with associated exuvia. Specimen label data and notes on habitats are provided. Antipredator defense structures and behaviour are noted for larvae and pupae of both species.

## Introduction

The Stenochiinae (Coleoptera: Tenebrionidae) are a large, diverse group of darkling beetles (Matthews et al. 2010), but even in areas with faunas considered to be well known, immature stages of many common species remain undescribed, especially the ephemeral pupae. Stenochiine larvae often possess distinctive apical abdominal armature, presumed to be defensive. Known pupal structures inspire equal curiosity and provide good characters for analysis of generic relationships (Bouchard and Steiner 2004). Larvae and pupae of *Glyptotus cribratus* LeConte from the southeastern United States and *Cibdelis blaschkei* Mannerheim from California – the type-species of their respective genera – are described in this study for the first time, with the intent of contributing to characterization of features useful for future identification and studies of phylogeny. Both species inhabit forested regions where adults, larvae and pupae have been found in soft rotten dry wood of dead branches on living trees or in sections recently fallen from them. The discovery and recognition of this particular niche should facilitate collection of these and other tenebrionid larvae occupying forest habitats.

## Materials and methods

Larval specimens were preserved in 80% ethanol; prior to this, some were killed either with hot water or by fumigation in ethyl acetate. Pupal specimens were similarly preserved as described earlier (Steiner 1995). Specimen label data below are given verbatim, with commas inserted for clarity, and breaks between labels are separated by a forward slash. Numbers of specimens bearing those data follow in parentheses, indicated as (L) larva, (P) pupa, or adult with associated exuvia. All specimens are deposited in the United States National Museum of Natural History (USNM), Smithsonian Institution, Washington, DC, USA.

## Systematics

### Glyptotus cribratus

LeConte

http://species-id.net/wiki/Glyptotus_cribratus

#### Background.

A single larva from southern Florida, USA, presumed to be *Glyptotus cribratus*, was reported by St. George (1924), who provided some key characters though the specimen’s identity has remained uncertain. New associated adult and larval material, including one larva reared to an adult and another providing the first known pupal specimen (described herein), shows that the larva from St. George’s account was not that of *Glyptotus*, as discussed below. The specimen, presumably in USNM, could not be located.

Three mature larvae, found in wood products from Mexico, 1982–84, were identified as “*Glyptotus* sp.” by T. J. Spilman, but it is uncertain what material he used to make this determination; these larvae are considered in the present study to be *Glyptotus cribratus*, identical to specimens from USA; the species is known from southern Texas and thus its occurrence in north-eastern Mexico is feasible.

#### Description of mature larva.

(Figs 1, 3–5, 9–12).

**Figures 1–8.**
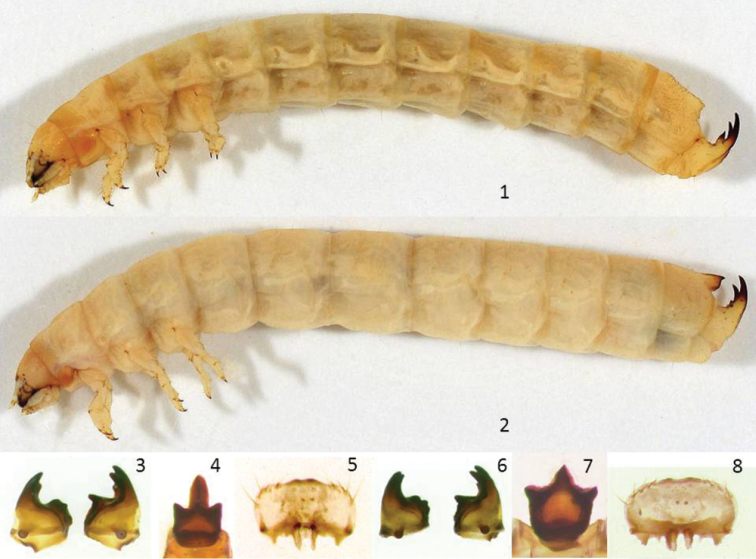
Mature larva of *Glyptotus cribratus*, lateral view, length 22 mm (**1**), mature larva of *Cibdelis blaschkei* lateral view, length 25 mm (**2**), *Glyptotus cribratus* larva, mandibles, ventral view, length 0.9–1.0 mm (**3**), epipharyngeal sclerome, length 0.4 mm (**4**), epipharynx, width 0.8 mm (**5**), *Cibdelis blaschkei* larva, mandibles, ventral view, length 0.7–0.8 mm (**6**), epipharyngeal sclerome, length 0.3 mm (**7**), epipharynx, width 0.7 mm (**8**).

**Figures 9–16.**
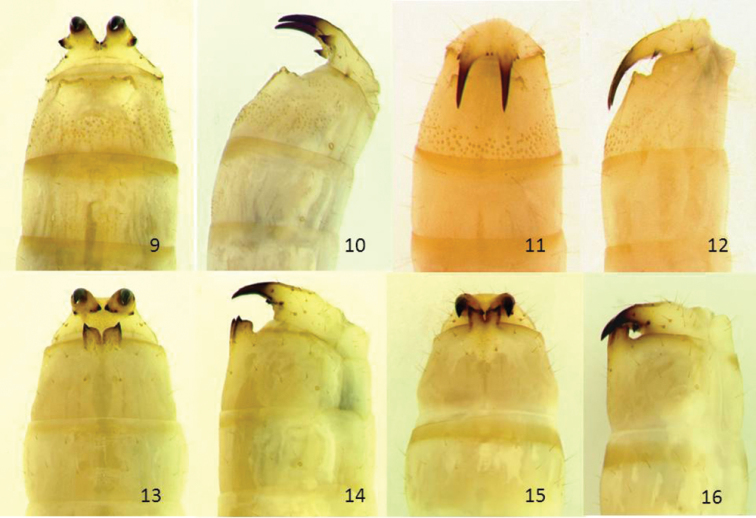
Abdominal apices of mature larvae of *Glyptotus cribratus* (**9–12**), dorsal (**9**) and lateral (**10**) view, urogomphi at rest, dorsal (**11**) and lateral (**12**) view, urogomphi raised, and abdominal apices of mature larvae of *Cibdelis blaschkei* (**13–16**), dorsal (**13**) and lateral (**14**) view, urogomphi at rest, dorsal (**15**) and lateral (**16**) view, urogomphi raised. Maximum widths of abdominal segments 3.3–3.5 mm.

**Body.** Length 23–30 mm; elongate-cylindrical, pale yellowish-white with light brown dorsal bands at posterior edges of terga and anterior edge of prothoracic tergum; mandible apices and bases, claws of tarsunguli, and apices of urogomphi and associated processes blackish to brown, heavily sclerotized; cuticle otherwise lightly sclerotized, surfaces shining, finely rugose, with scattered fine setae; abdominal tergum VIII slightly darker yellow-brown with scattered large circular punctures.

**Head.** Prognathous, head slightly declined, globular but slightly flattened dorsoventrally. Head capsule width 3.4–3.5 mm. Epicranial stem about one fifth head capsule length; frontal arms widely V-shaped, fine and obscure. Each half of head capsule with 10–13 scattered, long erect setae positioned dorsally and laterally. Stemmata five on each side closely posterior to antenna base, variably pigmented; anterior row of three closely spaced and usually darker than offset pair behind them. Clypeus convex, transverse, weakly trapezoidal, about two times wider than long, with one long seta on each side of disc and three smaller setae at lateral edges. Labrum transverse, convex, with two long discal setae, two anterior setae near midline, and four smaller, fine setae along each side of anterior edge. Epipharynx with three relatively stout setae along each side of anterior margin and two very short, stout medial spines, the pair slightly offset to left; with a cluster of 8–9 small round sensory papillae anterior to spines; tormae slightly asymmetrical. Antenna three segmented with membranous base globular, wider than long; first segment longest, cylindrical, wider toward apex, 2.5× longer than wide; second segment ovoid, two thirds as long as first, 2× longer than wide, with apical sensoria flat, kidney-shaped, partly encircling base of third segment; third segment very small, cylindrical, 1.5× longer than wide, with a single fine seta apically. Mandibles asymmetrical, apices tridentate, left mandible with a fourth feeble tooth dorsally along sharp incisor edge; left mola concave, with a prominent premolar tooth and three transverse, sclerotized ridges; right mola convex, with a transverse fossa surrounded by irregularly prominent ridges. Ligula with four fine apical setae; prementum, mentum, submentum each with a pair of long setae near base. Hypopharyngeal sclerome well developed, tridentate, with smooth concavity in middle; median tooth carinate, with Y-shaped arms to prominent, conical, lateral teeth; basal transverse ridge asymmetrical.

**Thorax.** Prothorax as long as wide; meso- and metathorax wider than long; terga with 9–12 fine setae on each side, more closely spaced laterally. Mesothoracic spiracle simple, ovate, slightly larger and narrower than abdominal spiracles; metathoracic spiracle visible, very small, nearly circular. Prothoracic leg slightly larger than mid- and hindlegs; all legs with trochanter elongated, with anterior and posterior rows of setae on ridges; femur and tibia bearing scattered, shorter setae; tarsungulus with two pre-basal setae; claw simple, sharp, curved apically, two thirds the length of tarsungulus.

**Abdomen.** Abdominal segments I-VII similar, nearly as long as wide, gradually slightly wider posteriorly; terga with sparse setae as on thoracic terga; spiracles annular, broadly ovate; sterna on each side with an anterior group of 4–5 setae of varying sizes and a pair of setae posteriorly. Tergum VII with a field of circular, deep punctures across anterior two thirds of middle. Tergum VIII as long as wide, abruptly narrowing posteriorly, with an extensive field of large, deep, circular punctures across anterior half and expanding on sides and dorsally with 7–8 scattered fine setae on each side; sloped posterior bearing two somewhat sclerotized, umbonate bullae on each side, large lateral one with a long seta arising from anterior of base of umbo; small umbo posterior to larger one and closer to midline, connected to larger by a feeble ridge, the four bullae forming a trapezoidal arrangement in posterior view, immediately anterior to a broad membranous apical area which opposes a similar dorsal membrane at anterior of tergum IX. Tergum IX short, about two thirds the width of tergum VIII and hinged to it, allowing curved urogomphi to come forward to oppose and contact bullae of tergum VIII; lateral hinge joint with a sclerotized, tooth-like, anterior process; urogomphi long, gradually tapered, divergent and curved dorsally with sharp apices pointing anteriorly, nearly round in cross-section, darkly sclerotized in apical half, each with three other sclerotized, tooth-like projections near base, as follows: large lateral claw-like process with apex pointed upward, forward and angled laterally, with a single dorsal seta below apex; small dorsal cone-like process bearing a single seta near apex; smaller, mesal, short, pointed to button-like process closely opposing other on opposite urogomphus. Other setae on tergum IX long, scattered; urogomphus with three setae on ventral (posterior) side; hinge process with a single seta near base; lateral and ventral surface with 7–8 scattered setae. Abdominal segment X small, ventral, transverse, semi-circular, convex, 3× wider than long, with a row of six fine setae across width; pygopods absent.

#### Description of pupa.

**Figures 17–20.**
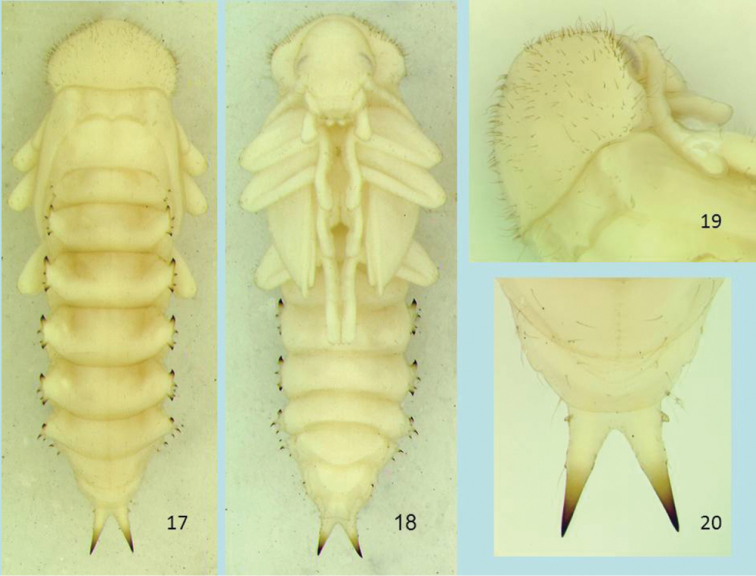
Pupa of *Glyptotus cribratus*, dorsal view (**17**) and ventral view (**18**), pronotum, dorsolateral view (**19**), abdominal apex and urogomphi, dorsal view (**20**). Length of pupa 15.1 mm.

(Figs 17–20, 25, 27).

**Body.** Length (from anterior edge of pronotum to tips of urogomphi) 15.1 mm, width of pronotum 3.9 mm; body color white with brownish surface setae, apices of urogomphi and spines on lateral processes; body very sparsely setose except pronotum with numerous fine discal and marginal setae, more dense anteriorly. Lateral processes of middle abdominal segments well developed, wing-like, bearing anterior and posterior smooth teeth and two smaller setigerous lateral spines between them; urogomphi long, smooth, gradually tapered to divergent, upturned apices.

**Head.** Hypognathous; surface smooth but with transverse wrinkles across frons; projection above antennal insertion rounded, not prominent; a few fine setae on frons, near eye, on clypeus and labrum; single seta on left mandible, near middle on outer curve; row of four setae on right mandible, from base to near middle on outer curve; single very small seta on outer edge of maxillary palpus, at base of last segment.

**Thorax.** Pronotum broadly shield-shaped, slightly wider than long, smooth with subtle transverse wrinkles; anterior angles and apex broadly rounded, posterior margin narrowly sinuate with posterior angles slightly pointed posteriorly. Pronotal surfaces with many short, fine setae except anterior margin and part of disc bare and setae more sparse in posterior part of disc; setae most closely spaced along margin of anterior angle, with some setae 2× longer than adjacent ones, fine and often curved; hypomeron smooth, with a few widely spaced fine setae; meso- and metatergite transverse, smooth, with very few fine setae; mesonotum produced and elevated posteriorly at middle (scutellar umbo); metanotum about 2× longer mesonotum, nearly 2× longer than abdominal tergite 1. Elytral sheath smooth with broad, shallow wrinkles; metathoracic wing sheath slightly shorter apically; meso- and metaventrite smooth. Legs and tarsi smooth, with a few scattered fine setae; femora with 5–7 setae from mid-length to near apex; tibiae with 3–5 setae along mid-length; protarsi with 3–4 setae on apical tarsomeres ventrally and laterally; apical tarsomeres of meso-and metatarsi with 1–2 setae laterally.

**Abdomen.** All surfaces smooth, bearing scattered fine setae; spiracles annular, vertically ovate to reniform, barely pigmented, visible on segments 2–6. Tergite 1 short, with five discal setae on each side, lateral process with single small posterior tooth and two small lateral spines with a wide, U-shaped emargination between them; smallest spine near base of tooth and bearing an apical seta, largest (anterior) sharply pointed, with a sub-apical seta. Tergites 2–5 of similar form, quadrate, transverse, with 4–5 discal setae on each side; lateral processes each with anterior and slightly smaller posterior teeth, stout but pointed at sclerotized, curved apices; lateral spines with sharp, sclerotized tips; larger of lateral spines near midpoint between teeth, bearing a sub-apical seta on posterior side, smaller spine arising from mid-length of posterior tooth, bearing a sub-apical seta on anterior side. Ventrites 2–6 smooth, convex, with 4–7 small fine setae on each side. Lateral process of tergite 6 with posterior tooth absent, both spines with sub-apical seta on posterior side. Tergite 7 narrower than preceding tergites, with four small fine setae on each side, with lateral process positioned and directed ventrally, not in same plane as preceding processes, anterior tooth reduced to a rounded lobe and posterior tooth absent, two posteriorly curved spines with sub-apical setae on posterior sides; sternite 7 roughly semicircular, transversely rugose apically, with three small discal setae on each side, the pair of larger setae along apical margin, with two smaller ones between them. Tergite 8 narrower than tergite 7, narrowing to broadly rounded apex, with lateral process reduced to a narrow ridge with two posteriorly curved spines only, posterior spine very reduced; spines with sub-apical setae on posterior sides; sternite 8 semicircular, with three small discal setae on each side, posterior most pair at sides of a transverse, raised area near apex. Tergite 9 short, bearing large divergent urogomphi, each gradually tapered and more sclerotized toward upturned, very sharp apices; cleft between urogomphi V-shaped with a narrowly rounded apex; base of urogomphus laterally with a single small, tooth-like protuberance, not heavily sclerotized, with a fine seta on mesal side near base; other long fine setae present on side of base of urogomphus and ventrolateral side of urogomphus to about mid-length, the latter arising from small protuberances. Genital segment (female pupa) roughly trapezoidal, smooth, with two divergent, protruding papillae bearing a single small seta laterally, papillae with a shallow V-shaped emargination between them.

#### *Glyptotus cribratus* larval, pupal and reared adult material examined.

**Specimens collected in USA.** “FLORIDA: Highlands Co., Archbold Biol. Sta., S of Lake Placid, S. side of Lake Annie, 27°12'35"N, 81°21'W, 19 April 2003 / In soft rotten dry wood of low dead branch on live Quercus virginiana; W. E. Steiner, J. M. Swearingen et al. collectors” (3 L); Same data except “Reared from larva; emerged October 2003, larval exuvia not recovered” (1 A); “FLORIDA: Highlands Co., Archbold Biol. Sta., S. of Lake Placid, forest tract, NE part, 27°12'N, 81°20'W, 19 April 2003 / W. E. Steiner & J. M. Swearingen collectors / In pithy rotten wood of dead standing oak branch, mixed scrub forest burned ca 1 year ago” (2 L); “FLORIDA: Highlands Co., Archbold Biol. Sta., S. of Lake Placid, hill area E of Station, 27°11'N, 81°20'30"W / 31 December 2006, W. E. Steiner, J. M. Swearingen, A. W. & B. B. Norden, collectors / In dry rotten wood of recently fallen dead branch of live oak” (1 L); “FLORIDA: Highlands Co., 2 km N. Cornwell at Kissimmee River, 1 March 1984 / In rotting wood of log of live oak / W. E. Steiner, A. G. Gerberich, J. E. Lowry collectors” (1 L); “GEORGIA: Camden County, Little Cumberland Island, 30°58'N, 81°25'W, 30 November 1997 / In dry soft rotten wood of hanging branch *Quercus virginiana* in maritime forest / W. E. Steiner, J. M. Swearingen, W. A. Dix, C. Wells collectors” (1 L); same data except “25 November 1998 / In dry soft rotten wood of dead branch in canopy of *Quercus virginiana* in maritime forest” (2 L); same data except “In pithy rotten wood of dead branch recently fallen from live oak, *Quercus virginiana*” (1 L); same data except “28 November 1998 / Associated with adult *Glyptotus cribratus* in dry soft rotten wood of small low branch on live *Quercus virginiana* in maritime forest” (1 L); “NORTH CAROLINA: Dare County, Kill Devil Hills, 35°59'33"N, 75°39'11"W, 23 February 2007, coll. W. E. Steiner & J. M. Swearingen / In dry rotten wood of recently fallen dead branch of s. red oak (*Quercus falcata*)” (1 L); same data except “Reared from larva in dry rotten wood of recently fallen dead branch of s. red oak (*Quercus falcata*); pupated 25 April, preserved 1 May 2007” (1 P with larval exuvia); “SOUTH CAROLINA: Dillon Co.; Fork; Little Pee Dee S.P., sand area, 34°19'10"N, 79°17'06"W, 16 April 2012 / In dry soft rotten wood of dead branch of live *Quercus laevis* in open pine-oak sand scrub; colls. J. C. Ciegler, W. E. Steiner, J. M. Swearingen” (1 L); “SOUTH CAROLINA: Georgetown County; Huntington Beach, near Murrells Inlet, 33°30'51"N, 79°03'09"W, 15 April 2012 / In dry soft rotten wood of dead lower branch *Quercus virginiana* in maritime forest; colls. J. C. Ciegler, W. E. Steiner, J. M. Swearingen” (1 L); “TEXAS: Comal County, Espinazo del Diablo, 9 km SW Wimberley, 29°55'30"N, 98°09'05"W, 17 November 2013 / In dry pithy wood of recently fallen branch of living *Quercus virginiana fusiformis* / Colls. W. E. Steiner, J. M. Swearingen, J. R. Ott, E. Silverfine” (1 L); “TEXAS: Hays County, Driftwood, at Dutchman Vineyards, 30°06'09"N, 98°0'51"W, 15 November 2013 / In dry pithy wood of dead low branches on large living *Quercus virginiana fusiformis* / Colls. W. E. Steiner, J. M. Swearingen, J. R. Ott, E. Silverfine” (4 L); “TEXAS: Hays County, Freeman Ranch, NW of San Marcos, 29°56'23"N, 98°0'44"W, 15 November 2013 / In dry pithy wood of dead low branch on large living *Quercus virginiana fusiformis* / Colls. W. E. Steiner, J. M. Swearingen, J. R. Ott, E. Silverfine” (1 L); same data except second label “In dry pithy wood of recently fallen branch of large living *Quercus virginiana fusiformis*” (1 L); “TEXAS: Hays County, Rutherford Ranch area NW of Kyle; oak grove near pond, 30°02'49"N, 97°57'56"W, 16 November 2013 / In dry pithy wood of recently fallen branch of large living *Quercus virginiana fusiformis* / Colls. W. E. Steiner, J. M. Swearingen, J. R. Ott, E. Silverfine” (1 L); same data except “(Preserved 11 Dec. 2013) / In dry pithy wood of dead low branch on large living *Quercus virginiana fusiformis*” (2L); “TEXAS: Hays County, Rutherford Ranch area NW of Kyle; near old ranch house ruins, 30°04'0"N, 97°56'37"W, 16 November 2013 / In dry pithy wood of recently fallen branch of large living *Quercus virginiana fusiformis* / Colls. W. E. Steiner, J. M. Swearingen, J. R. Ott, E. Silverfine” (1 L); “TEXAS: San Patricio Co., 12 km NE Sinton, Welder Wildlife Refuge, 8 December 1984, W. Steiner, B. Gill & D. Whitehead collrs. / In rotting wood of log of *Celtis* / larva coll. 8 Dec. 84, pupated 25 Apr. 85, eclosed 14 May 85, preserved 1 June” (1 adult pinned with larval and pupal exuvia); “VIRGINIA: City of Va. Beach, First Landing S. P., beach campground, 36°55.4'N, 76°2.8'W, 16 June 2007 / In pithy rotten wood of dead branch recently fallen from live oak, *Quercus virginiana*/ W. E. Steiner, J. M. Swearingen et al. collectors” (1 L).

**Specimens intercepted from MEXICO.**“*Glyptotus* sp. det. T.J. Spilman 1982, ex Mexico, at Hidalgo 4194, in *Prosopis* sp. stem, at Brownsville, #11491, 22v82, 82-6571” (1 L); “*Glyptotus* sp. det. T.J. Spilman 1983, ex Reynosa, Mexico, 8iii83, at Hidalgo 4194, on stem *Prosopis juliflora*” (1 L); “*Glyptotus* sp. det. T.J. Spilman 1984, ex Mexico, at Laredo, 17843, in rotting log, 4xii83, 84-549” (1 L).

### Cibdelis blaschkei

Mannerheim

http://species-id.net/wiki/Cibdelis_blaschkei

#### Background.

*Cibdelis blaschkei* is a very common beetle throughout much of California, with large series of adults represented in collections, but surprisingly, no specimens of its larvae or pupae could be found in museum holdings, nor are there any records of immature stages in the literature. The discovery of aerial dead wood larval habitats on trees in eastern USA localities led to the examination of similar wood in California, resulting in the collections listed below. Several other *Cibdelis* species have been described, all from California; the genus needs revision (Aalbu et al. 2002).

#### Description of mature larva.

Figs 2, 6–8, 13–16.

**Body.** Length 24–29 mm; elongate-cylindrical, pale yellowish-white with light brown dorsal bands at posterior edges of terga and anterior edge of prothoracic tergum; mandible apices and bases, claws of tarsunguli, and apices of urogomphi and associated processes blackish to brown, heavily sclerotized; abdominal terga VIII and IX dorsally slightly darker yellow-brown; prothoracic sternum in front of leg more sclerotized, light brown; cuticle otherwise lightly sclerotized, surfaces shining, finely rugose and obscurely punctate, with scattered fine setae.

**Head.** Prognathous, slightly declined, globular but slightly flattened dorsoventrally. Epicranial stem about one third head capsule length; frontal arms sinuate, lyre-shaped, fine and obscure. Each half of head capsule dorsally and laterally with 13–17 scattered, long erect setae. Stemmata five on each side closely posterior to antenna base, variably pigmented; anterior row of three contiguous and very close to offset pair behind them. Clypeus convex, transverse, weakly trapezoidal, about two times wider than long, with one long seta on each side of disc and three smaller setae at lateral edges. Labrum transverse, convex, with two long discal setae, two short anterior setae near midline arising from dark punctures, and four smaller fine setae along each side of anterior edge. Epipharynx with three relatively stout setae along each side of anterior margin and two very short, stout medial spines, the pair closely spaced and slightly offset to left; with a cluster of 7–8 small round sensory papillae anterior to spines; tormae slightly asymmetrical. Antenna three segmented with membranous base globular; first segment longest, cylindrical, narrower near middle, 3× longer than wide; second segment cylindrical, two thirds as long as first, 2× longer than wide, widest near apex, with apical sensoria flat, kidney-shaped, partly encircling base of third segment; third segment very small, cylindrical, 1.5× longer than wide, with a single fine seta apically. Mandibles asymmetrical, the right slightly smaller than left; right mandible with apex tridentate, palmate, left mandible with three broad apical teeth and a fourth pointed, thin, on sharp dorsal incisor edge; left mola concave, with a prominent premolar tooth and three transverse, sclerotized ridges; right mola convex, with two transverse fossae surrounded by irregularly prominent ridges. Ligula with six small apical setae arranged in two rows; prementum, mentum, submentum each with a pair of long setae near base. Hypopharyngeal sclerome well developed, tridentate, with smooth concavity in middle; median tooth with V-shaped carinae, arms forming a bridge to prominent crest of lateral teeth; basal transverse ridge symmetrical, concave across middle.

**Thorax.** Prothorax as long as wide; meso- and metathorax about 2× wider than long; protergum with 11–15 fine setae of varying size on each side, sparsely arranged in two bands; meso- and metaterga with 6–7 similar scattered setae. Mesothoracic spiracle simple, irregularly ovate, slightly larger and narrower than abdominal spiracles; metathoracic spiracle not visible. Prothoracic leg slightly larger than mid- and hindlegs; all legs with trochanter elongated, with anterior and posterior ridges bearing a few fine setae; femur and tibia bearing scattered, shorter setae; tarsungulus with two pre-basal setae; claw simple, sharp, curved apically, two thirds the length of tarsungulus.

**Abdomen.** Abdominal segments I-VII similar, slightly wider than long, successive segments gradually slightly wider posteriorly; terga with sparse long setae as on thoracic terga, 9–10 on each side; spiracles annular, broadly ovate; sterna on each side with an anterior row of three fine setae and a pair of setae posteriorly. Tergum VIII nearly as long as wide, slightly narrowed posteriorly, dorsally with 9–10 scattered fine setae on each side, those nearest dorsal process arising from circular, pigmented punctures and in a moderately sclerotized area with smaller scattered punctures; dorsal outline in lateral view straight from base to apex of pointed, posterior process, one on each side, the close pair in dorsal view forming a V-shaped cleft at midline; pointed apices divergent, darkly sclerotized, joined to rounded mola-like process ventral and mesal to them; dorsum behind processes abruptly sloped downward to a broad membranous apical area that opposes a similar dorsal membrane at anterior of tergum IX. Tergum IX short, about two thirds the width of tergum VIII and hinged to it, allowing curved urogomphi to come forward to oppose and straddle pair of processes of tergum VIII; lateral hinge joint obscure, without sclerotized, tooth-like, anterior process; urogomphi robust, gradually tapered, slightly divergent and curved dorsally with sharp apices pointing anteriorly, nearly round in cross-section, darkly sclerotized in apical half, each with sclerotized, tooth-like projections near base, as follows: small dorsolateral conical process without associated seta; dorsomedial process bearing a single small seta between larger pointed mesal tooth and feeble lateral tooth, the mesal teeth closely opposing each other between urogomphi. Other setae on tergum IX long, scattered, those on and near base of urogomphus arising from large, circular, pigmented punctures; urogomphus with a lateral seta near mid-length, two on ventral (posterior) side; lateral and ventral surface with 9–12 scattered setae of varying size. Abdominal segment X small, ventral, transverse, semi-circular, convex, 2.5× wider than long, with a row of 6 fine setae across width; pygopods absent.

#### Description of pupa.

**Figures 21–24.**
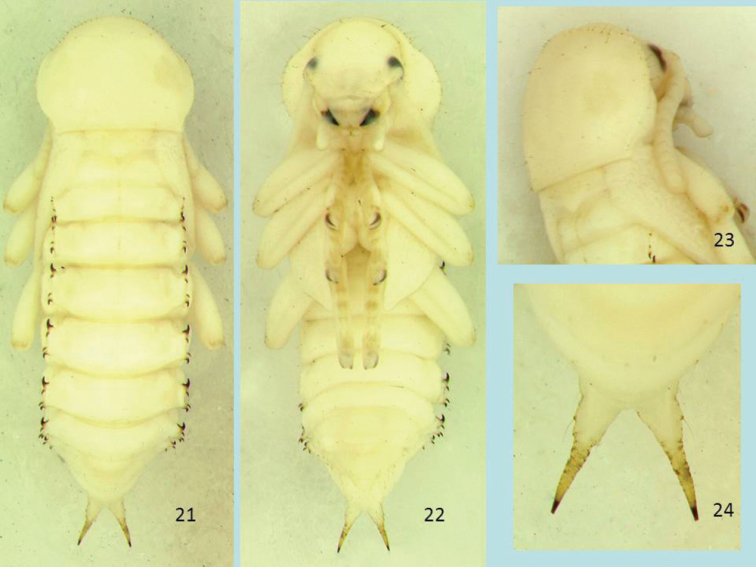
Pupa of *Cibdelis blaschkei*, dorsal view (**21**) and ventral view (**22**), pronotum, dorsolateral view (**23**), abdominal apex and urogomphi, dorsal view (**24**). Length of pupa 16.2 mm.

(Figs 21–24, 26, 28).

**Figures 25–28.**
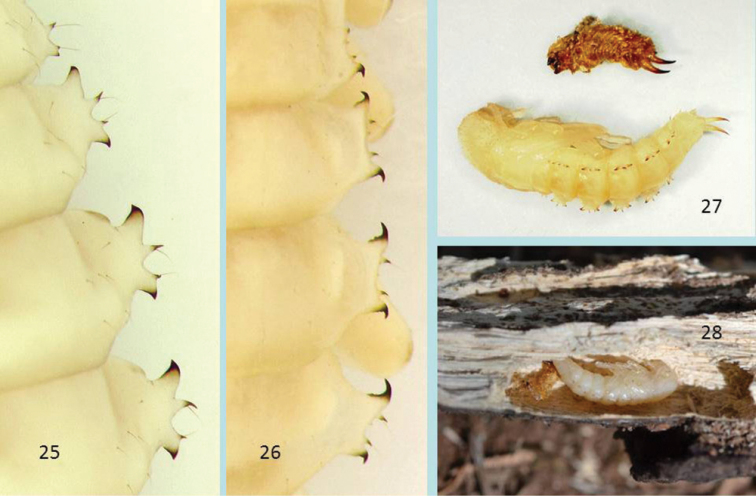
Right lateral abdominal processes of tergites 1–4, pupa of *Glyptotus cribratus* (**25**) and *Cibdelis blaschkei* (**26**), *Glyptotus cribratus*, live pupa (dorsolateral view) and associated larval exuvia, Kill Devil Hills, North Carolina (**27**), *Cibdelis blaschkei*, live pupa (lateral view) and associated larval exuvia in pupal cell of oak wood, Angwin, California (**28**). Length of pupae 15–16 mm.

**Body.** Length (from anterior edge of pronotum to tips of urogomphi) 15.7–18.5 mm, width of pronotum 4.8–5.0 mm; body color white with brownish surface setae, apices of urogomphi and spines on lateral processes; body very sparsely setose. Lateral processes of middle abdominal segments well developed, wing-like, bearing anterior and posterior curved teeth and two smaller setigerous lateral spines between them; urogomphi long, wrinkled at base, gradually tapered to divergent, sharp apices.

**Head.** Hypognathous; surface with dense shallow punctures and between eyes with faint transverse wrinkles; projection above antennal insertion absent; a few fine setae on frons, near eye, on clypeus and labrum; a row of four setae on each mandible from base to apical one-third along outer curve; single very small seta on outer edge of maxillary palpus, at base of last segment.

**Thorax.** Pronotum broadly shield-shaped, slightly wider than long, smooth with a mixture of fine shallow punctures and subtle transverse wrinkles; anterior margin broadly rounded, lateral margin slightly explanate with thick bead, posterior margin nearly straight with posterior angles narrowly rounded, not produced posteriorly. Pronotal surfaces with sparse, fine setae along anterior and lateral margin, much smaller across middle; most of disc bare except for widely spaced setae, three on each side anteriorly, two posteriorly, and 1–3 very small setae laterally; hypomeron smooth, with a few widely spaced fine setae, some larger immediately under lateral margin; meso- and metanotum transverse, smooth, with 1–2 fine setae on each side; mesonotum produced and slightly elevated posteriorly at middle (scutellar umbo); mesonotum, metanotum, and abdominal tergite 1 short, roughly equal in length. Elytral sheath smooth with feeble, longitudinal furrows and sub-apical raised bulla; metathoracic wing sheath of thin membrane and shorter than elytral sheath, not visible beneath elytral sheath (unless dissected or observed in bloated specimens); meso- and metasternum short, smooth. Legs and tarsi smooth, with a few scattered fine setae; femora with 7–10 setae from mid-length to near apex; tibiae with 3–5 setae along mid-length; protarsi with 5–6 setae on apical tarsomeres ventrally and laterally; apical tarsomeres of meso-and metatarsi with 3–4 setae laterally.

**Abdomen.** All surfaces smooth, bearing scattered fine setae; spiracles annular, rounded, barely pigmented, visible on segments 2–6. Tergite 1 short, with 4–5 discal setae on each side, lateral process with anterior tooth reduced to a small conical projection bearing an apical seta; posterior tooth with sharp, sclerotized apex abruptly directed posteriorly; lateral spine near base of each tooth small, narrowly conical, lightly sclerotized, directed slightly to anterior, with a broad, shallow emargination between them; anterior spine with seta directed posteriorly; posterior spine with seta directed anteriorly. Tergites 2–6 of similar form, quadrate, transverse, with 4–5 discal setae on each side; lateral processes each with prominent anterior and slightly smaller posterior teeth, strongly curved and gradually tapered to sharp, sclerotized apices; anterior tooth with small serrations along curve on anterior side; each tooth with associated lateral spine arising near base between them and with apex curved posteriorly; spines bearing a long seta on dorsal side, arising from a sclerotized elevated base; a broad, shallow emargination between spines with a very small setiferous accessory spine along length, usually closer to anterior spine; anterior spine with seta directed posteriorly; posterior spine with seta directed anteriorly in most examples. Ventrites 2–6 smooth, convex, with 1–7 small fine setae on each side, basal ventrites with fewer setae. Tergite 7 narrower than preceding tergites, with 3–4 small fine setae on each side, anterior tooth small, posterior tooth absent, posterior spine very small, accessory spine absent; sternite 7 semicircular, with 3–4 small discal setae on each side, the larger setae paired near apical margin. Tergite 8 narrower than tergite 7, narrowing to broadly rounded apex, with lateral process reduced to a narrow ridge with 2 posteriorly directed setae only; discal setae 3–4 on each side; sternite 8 semicircular, with 2 small discal setae on each side near apical margin. Tergite 9 small, narrow, bearing large, divergent, posteriorly directed urogomphi, each gradually tapered and more sclerotized toward sharp apices, with irregular crenulate surface along mid-length; cleft between urogomphi V-shaped with a narrowly rounded apex; base of urogomphus laterally with a single prominent seta arising from a sclerotized, raised base; 7–8 other fine setae present ventrally on base of urogomphus and ventrolateral side of tergite 9; sternite 9 in male pupae small, narrowly transverse, with 2 setae on each side; sternite 9 in female pupae not visible. Genital segment in male pupae small, recessed, smooth, convex, slightly wider than long, with rounded apical lobes separated with a small median notch; in female pupae large, produced, smooth, roughly trapezoidal, with two divergent papillae bearing a single small seta laterally, papillae separated by a sinuate apical margin.

#### *Cibdelis blaschkei* larval, pupal and reared adult material examined.

“CALIFORNIA: Contra Costa Co., Tilden Park NE of Berkeley, 37°53'24"N, 122°14'13"W, 23 June 2012, colls. W. E. Steiner, J. M. Swearingen et al. / Under bark of fallen pine branch in mixed forest grove” (1 P); “CALIFORNIA: Napa Co., Angwin, near airport, 38°34'13"N, 122°25'50"W, 29 June 2012, coll. W. E. Steiner & J. M. Swearingen, In rotten dry wood of fallen oak branch in mixed forest” (5 L); same data except: “preserved 2 July 2012” (1 P); “preserved 4 July 2012” (2 P); “preserved 9 July 2012” (2 P); “CALIFORNIA: Napa County, St. Helena, 16 Feb. 2003, W. E. Steiner, J. M. Swearingen et al. collectors / In dry rotten wood of recently fallen dead branch of oak” (2 L); same data except “21 Dec. 2003” (2 L); “CALIFORNIA: Napa County, 7 km NW St. Helena, 38°32'N, 122°31'W / 15 Dec. 2003, W. E. Steiner & J. M. Swearingen collectors / In dry rotten wood of recently fallen dead branch of oak” (2 L); “CALIFORNIA: Napa Co., Silverado area, 5 km NE of Napa, 38°20'N, 122°15'W / 18 Feb. 2003, W. E. Steiner, J. M. Swearingen collectors / In pithy dry wood of fallen oak branch ca. 8 cm diameter on ground, open hills with oak groves (3 L); same data except “25 April 2004” and “branch ca. 5 cm.” (1 L); “CALIFORNIA: Napa Co., Skyline Park area, 5 km SE of Napa, 38°16'N, 122°15'W / 8 Feb. 2003, W. E. Steiner, J. M. Swearingen collectors / In dry rotten wood of recently fallen dead branch of oak” (3 L); “CALIFORNIA: Napa Co., Soda Canyon Road at ravine, NE of Napa, 38°23'06"N, 122°16'51"W, 22 June 2012, In rotten dry wood of fallen oak branch, roadside in open forest” (1 P, partially eaten, larval exuvia not found); “CALIFORNIA: Napa Co., Soda Springs Road NE of Napa, 38°23'28"N, 122°17'07"W, 22 June 2012, coll. W. E. Steiner & J. M. Swearingen, In rotten dry wood of fallen oak branch, roadside in open forest” (1 L); same data except “28 June 2012” (1 L); “CALIFORNIA: Napa Co., Spanish Flat, near Lake Berryessa, 11 April 2008 / pupated 21 April, preserved 29 April 2008 / W. E. Steiner, J. M. Swearingen collectors, In rotten wood of fallen branch of oak on ground (larva)” (1 P); “CALIFORNIA: Napa Co., Yountville, 38°24'N, 122°22'W / 9 Feb. 2003, W. E. Steiner, J. M. Swearingen collectors / In rotten dry wood of fallen branch of *Quercus garryana* among vineyards” (1 L); same data except “In rotten dry wood of recently fallen dead branch of *Quercus garryana* / Reared from larva found 9 Feb. 2003, pupated 23 May (found), preserved 1 June 2003” (1 P); “CALIFORNIA: Napa Co., 3 km NW Yountville, 38°25'01"N, 122°23'58"W, 21 October 2010, coll. W. E. Steiner & J. M. Swearingen / In rotten dry wood of fallen branch *Quercus garryana* among vineyards” (1 L); same data except “38°25'05"N, 122°23'52"W, 25 June 2012 / Found in pupal cell in rotten dry wood of fallen branch *Quercus garryana* among vineyards; (1 P); same data except “preserved 19 July 2012” (3 teneral adults pinned with associated larval and pupal exuvia in gelatin capsule). “CALIFORNIA: Sonoma Co., 1 km N Wikiup, 38°31'33"N, 122°45'41"W, 15 October 2009, coll. W. E. Steiner & J. M. Swearingen / In dry rotten wood of recently fallen dead branch of oak” (5 L).

#### Habitats and observations on life history.

Both *Glyptotus cribratus* and *Cibdelis blaschkei* are beetles of forested areas, their larvae being dependent on dead, rotten wood for survival. They tend to be more common in forest edge habitats or at single trees or groves in open areas, where wood dries out more rapidly and is slower to decay than in mesic forest interiors. These species may be avoiding attack by fungal, bacterial, or other pathogens and/or avoiding competition from insects inhabiting more damp wood in shaded situations. Furthermore, larvae are rarely if ever found in rotten wood on the ground, with the exception of recently fallen branches; these beetles appear to be specialists in dead wood involving mostly smaller branches, on living, usually old trees. Specimen data and observations indicate that if inhabited branches happen to fall, older, nearly full-grown larvae may be able to complete development. Adults probably breed commonly in canopy-level wood; an opportunity to observe *Glyptotus cribratus* on dead canopy branches of oak (Little Cumberland Island, Georgia) led to collection of adults and larvae in exposed, rotten branches several meters above ground. The dead oak branches in which *Glyptotus* and *Cibdelis* have been found were usually covered with lichens, which possibly serve as food for adults.

Adult beetles are nocturnal, often found on bark at night, but hide during the day in hollow dead branches as well as under the bark of dead branches or main trunks. Adults and larvae of both species are active throughout the year, but pupation seems to be restricted to spring and summer. Larvae tunnel in moderately soft dry wood, consuming it (and probably fungal tissue within) and depositing pelleted frass in the burrow; they can occur immediately under the bark or in the branch interior. Most larvae have been collected in smaller branches, 3–15 cm diameter, with wood that is easily broken apart by hand. Pupation occurs in the same wood. In one instance, the pupal period lasted 19 days for a Texas specimen of *Glyptotus*, reared at 22–26°C; no comparable pupal data are available for *Cibdelis*, but the period is likely similar. Except for one record of a pupa beneath bark of an undetermined pine species, *Cibdelis blaschkei* is typically associated with oaks.

*Glyptotus cribratus* occurs from coastal Virginia to Texas (Hoffman et al. 2002) where it is often associated with live oak, *Quercus virginiana* Mill., which has a similar distribution, but other species of *Quercus*, as well as *Celtis* and *Prosopis* spp., are recorded hosts. Beetles are most common in maritime oak forests and sandhill habitats of the coastal plain but also occur in middle elevations of the southern Appalachians. This species is common as well in the elevated karst areas of central Texas (Edwards Plateau), which have a distinct oak flora.

#### Observations on defense structures and behaviour.

**Larval characters.** In larvae of both *Cibdelis* and *Glyptotus*, the manner in which the long, upcurved urogomphi oppose the raised areas and posterior projections of tergite 8 appear to form a pinching structure, as seen in occasional specimens preserved in the “closed” position (Figs 11, 12, 15, 16). The opportunity to observe pinching behavior was offered by the recent collections of larvae on several occasions. When larvae are removed from the burrow in dead wood, they appear incapable of rapid evasive movement but do writhe in a circular movement when held at the middle by forceps or fingers, and the hinged urogomphi can be seen to open and close against segment 8 when the end of the abdomen is touched. Inserting a stiff hair, fine piece of grass, or insect pin tip in the dorsal gap between tergites 8 and 9 usually prompted the larva to pinch; occasionally, the larva will hold on and can be lifted off the substrate for several seconds before releasing the pinch. When full-grown larvae of *Glyptotus* were first exposed and restrained, pinches were observed and felt on fingertips; the larvae also appeared to be trying to bite with mandibles. These actions could possibly defend the larvae from attack by small lizards and other predators. When approached in the wood tunnel by a potential predatory insect, either end could be capable of some defense.

Pointed, upturned urogomphi are present in many tenebrionid larvae, but few posess opposing processes on tergite 8 and the hinge-like joint between segments. Long tactile setae, “trigger hairs,” are associated with processes on tergite 8 and occupy the space between the sloped dorsum of tergite 8 and the urogomphi at rest. The paired processes seen in *Cibdelis* larvae are of unique form, previously unknown, but the hinge-like joint is not apparent. The raised bullae of tergite 8 and other features of the pinching assembly in *Glyptotus* are very similar to those of the related stenochiine larva, *Haplandrus fulvipes* (Herbst) (St. George 1924, Figs 38–39). In the same work, a larva identified as *Glyptotus* from Florida was described in the key with “Pygidium with transverse row of strong, hook-shaped, seta-bearing spines anterior to cerci” but was not illustrated. This larva could not have been *Glyptotus* because these different features are all on tergum 9; no modifications of tergum 8 were noted.

Larvae of Helopini, also described from dead wood, have abdominal apices very similar to those of *Glyptotus*, for example *Helops caeruleus* L. (Schiödte 1878, Figs 20–21, Plate 11) and *Deretus spinicollis* Schawaller (Purchart and Nabozhenko 2012, Figs 9–10). The pinching ability of these larvae is probably comparable to that of *Glyptotus*.

**Pupal characters.** Like known pupae of other stenochiines, those of *Cibdelis* and *Glyptotus* are armed with lateral abdominal gin traps that pinch between the posterior and anterior curved teeth, with lateral projecting spines and associated tactile hairs. Use of these structures and associated body movements have been studied in other pupae (Bouchard and Steiner 2004 and papers cited within) and the live pupae observed in this study displayed these actions. Pupal cells in the soft wood (Fig. 28), formed by the mature larva before pupation, offer a large space in which pupae can actively rotate the body and use pinching organs. As noted in other stenochiine pupae, gin trap teeth of opposing lateral processes have the “posterior is ventral to anterior” closure type (Steiner 1995, Fig. 104) in the pinched position.

Pupae of *Glyptotus cribratus*, a winged species, possess fully formed wing sheaths beneath the elytral sheaths. Conversely, *Cibdelis blaschkei* is wingless, with elytra partially fused, though pupae still retain empty wing sheaths of nearly full length—a condition noted by Spilman (1979) in which a long sheath with vestigial or absent wing development indicates occasional flightlessness within groups otherwise characterized by flight. This is the case among Stenochiinae, where flightlessness appears to have evolved multiple times within a large clade of mostly winged species (Matthews et al. 2010).

## Supplementary Material

XML Treatment for Glyptotus cribratus

XML Treatment for Cibdelis blaschkei
